# Zenker’s diverticulum in the era of third-space endoscopy: innovations, challenges, and adverse event management

**DOI:** 10.1016/j.igie.2026.01.011

**Published:** 2026-01-27

**Authors:** Dimo Dimitrov, Dennis Yang, Peter V. Draganov

**Affiliations:** 1 Department of Medicine, Boston Medical Center – Brighton, Boston, Massachusetts, USA; 2 Department of Gastroenterology, Advent Health, Orlando, Florida, USA; 3 Division of Gastroenterology, Hepatology and Nutrition, University of Florida, Gainesville, Florida, USA

## Abstract

Endoscopic management of Zenker’s diverticulum has rapidly evolved with the emergence of innovative therapeutic endoscopic approaches and the integration of novel devices. These techniques offer unprecedented precision and safety, although rare, adverse events can become catastrophic if not promptly recognized and managed. This narrative review explores recent innovations in third-space techniques and novel devices, with a focused, detailed discussion on recognizing and effectively managing adverse events.

## Introduction

Zenker’s diverticulum (ZD) is a posterior pharyngoesophageal outpouching, predominantly affecting older adults,^1^ with a prevalence ranging from 0.01% to 0.11%.^2^^,^^3^ It can significantly impair patient quality of life because of symptoms such as dysphagia, regurgitation, aspiration, halitosis, and unintended weight loss.^2^ Treatment is indicated for symptomatic ZD,^4^ regardless of size, to relieve symptoms, prevent adverse events such as poor nutrition and aspiration pneumonia, and correct the pouch, which has the tendency to increase in size.^5^

Open surgical cricopharyngeal myotomy with diverticulectomy was historically the reference standard for ZD, offering success rates above 90%.^6^^,^^7^ However, it carries significant morbidity because of its invasiveness and extensive neck dissection, as well as longer recovery and a small but present mortality risk (1%-2%).^2^^,^^6^^,^^8^

In recent decades, minimally invasive transoral endoscopic approaches have largely replaced open surgery because of lower risk and faster recovery.^9^ Rigid endoscopic stapling has excellent short-term clinical success (85%-95%) but recurrence can be as high as 15%.^10^ Furthermore, anatomical limitations related to poor visualization, limited neck mobility, and small diverticulum size can lead to procedure abandonment in about 9% of cases,^10^ typically requiring conversion to open surgery.^11^^,^^12^

Flexible endoscopic septotomy (FES), another minimally invasive alternative, offers high initial efficacy and safety of around 90%.^5^^,^^13^ However, it has notable recurrence rates up to one-third of cases in some studies,^14^^,^^15^ requiring multiple sessions,^15^^,^^16^ attributed to incomplete septotomy.^16^ Traditionally, the septal division is carried out to the lower distal extent of the diverticulum but not beyond, to avoid perforation into the mediastinum. As a result, a small portion of the septum remains intact and is believed to be the main reason for diverticulum recurrence. This has driven the development of innovative endoscopic techniques and devices to improve efficacy and reduce recurrence while maintaining safety.

## Newer endoscopic techniques and devices

### The rise of third-space endoscopy

#### Zenker’s peroral endoscopic myotomy

Zenker’s peroral endoscopic myotomy (Z-POEM) is a more recent innovation that uses principles of third-space endoscopy, specifically derived from its direct ancestor, esophageal peroral endoscopic myotomy (E-POEM).^17^ The procedure involves the following steps: (1) a longitudinal mucosal incision on the posterior pharyngeal wall, 2 to 3 cm above the diverticulum; (2) submucosal tunneling down along both sides of the diverticular septum (esophageal and diverticular sides); (3) full-thickness myotomy of the cricopharyngeal muscle and septum; and (4) closure of the mucosal entry with endoscopic clips.^2^^,^^18^ Importantly, the original Z-POEM technique and many variations targeted only the cricopharyngeal and septal muscle but preserved the mucosal component of the diverticulum.

Z-POEM has consistently demonstrated high clinical efficacy with initial success rates above 90%.^2^^,^^16^^,^^19^^,^^20^ In a single-center series, all patients experienced symptom improvement, without the need for repeat myotomy in the short-to-intermediate term.^12^ Moreover, Z-POEM demonstrates excellent results in managing both small (<2 cm) and large (>4 cm) ZDs,^21^^,^^22^ as well as in salvage recurrence cases with fibrotic septa.^2^

Z-POEM has rapidly gained adoption as accumulating data continue to support its efficacy and safety. A recent meta-analysis (7 studies, 580 patients) found that although Z-POEM and flexible septotomy have similar technical success (∼99%), Z-POEM achieves significantly higher clinical success (risk ratio ≈ 1.11; *P* = .001) with no statistically significant differences in recurrence or adverse events^2^^,^^23^ (Table 1). Nevertheless, it is important to note that all the published studies are observational, and high-quality randomized controlled trials are lacking. Moreover, given its broad availability and comparable outcomes, the European Society of Gastrointestinal Endoscopy continues to recommend FES as first-line therapy for symptomatic ZD.^24^ The main conceptual advantage of Z-POEM is that the myotomy can be extended beyond the distal extent of the diverticulum, thus potentially decreasing the risk of recurrence. Importantly, the technique of Z-POEM is not standardized, and multiple variations have been described, making comparative studies difficult to interpret.

**Table 1:** Comparison of treatment modalities for Zenker’s diverticulum

Treatment modality	Invasiveness	Reported clinical success (%)	Reported recurrence rate (%)	Key limitations and adverse events
Open surgery	High	>90%	3.6%-10%	Significant perioperative morbidity; extensive neck dissection required; prolonged recovery period; mortality risk 1%-2%
Rigid endoscopic stapling	Moderate	85%-95%	5%-15%	Anatomical limitations result in ∼9% procedure abandonment rate; require general anesthesia; limited applicability in small diverticula
Flexible endoscopic septotomy	Low	75%-81.8%	13%-17.1%	Higher recurrence rate secondary to incomplete myotomy; may require multiple treatment sessions; limited efficacy in large diverticula
Z-POEM and other ESTT methods	Low	87.4%-92.1%	7.8%-10.3%	Requires specialized third-space endoscopy expertise; prolonged procedure duration, particularly during the learning curve; limited long-term data

*ESTT*, Endoscopic submucosal tunneling technique; *Z-POEM*, Zenker’s peroral endoscopic myotomy.

#### Z-POEM technique evolution

Because experience with submucosal endoscopy has grown, several technical modifications have been introduced: (1) the mucosal entry above the diverticulum has been largely abandoned, and direct transverse incision over the septum is favored; (2) variations in the tunneling approach include, for instance, peroral endoscopic septotomy, in which the mucosal entry is created directly over the septum for more direct access; (3) “tunneling-free” Z-POEM, which uses a direct mucosal incision over the septum followed by limited submucosal dissection^25^; and (4) other named variations, such as modified Z-POEM,^26^ “noninjection nontunnel” Z-POEM (NiZ-POEM),^27^ endoscopic transversal incision and longitudinal septostomy,^28^ and peroral endoscopic diverticulotomy,^29^ further illustrate the flexibility and ongoing evolution of endoscopic myotomy for ZD.^17^

Furthermore, Z-POEM has been adapted for challenging anatomies. In patients with very large diverticula, Z-POEM with mucosotomy of the septal bridge can be performed after the myotomy to prevent a persistent reservoir effect and reduce regurgitation.^17^ For recurrent ZD with significant fibrosis from prior interventions, a hybrid Z-POEM can be used, combining an initial septotomy to cut through scar tissue with submucosal tunneling once a clear tissue plane is reached.^17^

#### Advances in septotomy devices

Traditionally, the needle-knife (typically an endoscopic retrograde cholangiopancreatography knife for precut sphincterotomy) has been used for standard septotomy. As a freehand cutting tool that heavily relies on precise hand–eye coordination and makes controlling incision depth difficult, it carries a risk of inadvertent perforation.^8^ To address these safety concerns, newer devices have emerged to provide superior precision, control, and efficacy (Table 2).

**Table 2:** Endoscopic devices used in Zenker’s diverticulum treatment

Device	Mechanism	Primary application	Notable advantages
HookKnife (Olympus America, Center Valley, Pa, USA)	Rotatable 5-mm hook tip that lifts tissue and then cuts	FES; occasionally for Z-POEM tunnel entry	Provides precise dissection by lifting muscle fibers before cutting; reduces deep thermal injury risk; achieves high clinical success rates
Stag Beetle Knife (Olympus America)	Monopolar, rotatable scissor-type forceps that grasp tissue, coagulate, and cut	FES and peroral myotomy	Controls incision thickness; insulated blades with 360° rotation provide stable, precise positioning; simultaneously coagulates vessels to minimize bleeding
ClutchCutter (Fujifilm, Tokyo, Japan)	Insulated scissor-type knife that can grasp and electrosurgically cut tissue	FES; nontunneling Zenker’s POEM (NiZ-POEM)	Grasp-and-cut action resembles the bite biopsy technique; allows tissue regrasping to prevent miscutting 360° jaw rotation with simultaneous cutting and coagulation
Triangle Tip Knife (Olympus America)	Triangular tip with integrated water-jet nozzle and 3 L-shaped hooks	Submucosal tunnel creation and cricopharyngeal myotomy (Z-POEM)	Water-jet provides injection and spray-coagulation functions; 3-directional hooks enable accurate muscle fiber grasping without instrument rotation; integrated hemostasis reduces accessory requirements
HybridKnife (Olympus America)	Combined high-pressure water jet and electrosurgical blade	Submucosal tunneling procedures, including Z-POEM	Enables submucosal elevation and incision with a single instrument; alternating injection and cutting without device exchange saves time and reduces perforation risk

*FES*, Flexible endoscopic septotomy; *NiZ-POEM*, noninjection nontunnel Zenker’s peroral endoscopic myotomy; *POEM*, peroral endoscopic myotomy; *Z-POEM*, Zenker’s peroral endoscopic myotomy.

#### Needle-type knives

Among newer needle-type knives, the Triangle Tip Knife (TT Knife, Olympus America, Center Valley, Pa, USA) and HookKnife (Olympus America) represent refinements adapted from the esophageal E-POEM technique and endoscopic submucosal dissection, respectively. The TT Knife facilitates submucosal tunneling but is hindered by its bulky profile, limiting its maneuverability in the hypopharynx. By contrast, the Hook Knife's rotatable, “hook-and-cut” maneuver elevates muscle fibers before transection, enhancing depth control and reducing collateral thermal injury.^30^ Limitations include a steep learning curve because maneuvering the sharp, rotatable tip in the confined hypopharynx is technically demanding and requires considerable expertise. Safe use also relies heavily on the assistant's experience and skill in rotating and maintaining the knife's orientation. Both devices have demonstrated high technical success rates with few minor adverse events.^10^

Several other devices integrate injection and cutting into a single platform, improving procedural efficiency by minimizing exchanges. The HybridKnife (Erbe, Tübingen, Germany) combines an electrosurgical blade with a high-pressure water jet that provides excellent injection performance; however, it requires dedicated capital equipment (electrosurgical water-jet system [dedicated equipment for HybridKnife]). Other needle-type knives with injection capabilities can also be used for Z-POEM, including the DualKnife J (Olympus America), FlushKnife (Fujifilm, Tokyo, Japan), ORISE ProKnife (Boston Scientific, Marlborough, Mass, USA), and GoldKnife (Micro-Tech, Ann Arbor, Mich, USA).^8^

#### Scissor-type knives

Scissor-type knives represent a major conceptual advance by allowing tissue to be grasped with their opposing blades, coagulated, and then transected under direct control.^31^^,^^32^ The Stag Beetle Knife (SB Knife) (Olympus America) is available in multiple jaw sizes: the standard (7-mm jaw), the SB Knife Junior (3.5-mm blade for confined spaces),^30^ and the GX model with serrated edges for improved grip on fibrotic tissue.^30^ It provides secure tissue capture, 360° rotation, and reliable hemostasis,^31^ which is especially valuable when navigating fibrotic or challenging anatomy.^33^ A 2024 meta-analysis of 7 studies (n = 268) evaluating the SB Knife across various techniques, including endoscopic septotomy and diverticulectomy, reported approximately 98% technical success, initial symptom improvement in about 88% (rising to 96% with retreatment), and pooled minor adverse events in 13.2% intraprocedurally and 9.3% postprocedurally, with no severe events.^34^

The ClutchCutter (Fujifilm), with serrated and insulated jaws (3.5 or 5 mm) that can rotate 360°, offers similar grasp-and-cut functionality while minimizing collateral thermal injury.^32^ Its use has facilitated innovations such as NiZ-POEM,^27^ with a recent case series (n = 20) reporting 100% technical and clinical success and mean procedure times around 30 minutes.^35^ Comparative reports suggest the ClutchCutter may reduce bleeding, shorten procedure duration, and lower the need for repeat sessions compared with needle-type devices.^32^

The SpydrBlade Flex system (Creo Medical, Chepstow, United Kingdom) is a recent addition, combining advanced bipolar radiofrequency cutting with microwave coagulation, enabling simultaneous dissection and hemostasis. This is a relatively new device with no comparative data; however, it offers the theoretical advantage of using bipolar energy.

#### Comparative perspective

Overall, needle-type knives remain familiar and effective but require advanced operator skill and carry higher risks of uncontrolled depth. In contrast, scissor-type knives enhance procedural safety through grasp-and-cut mechanics, consistent hemostasis, and shorter procedure times, making them increasingly favored for both primary and salvage cases, particularly in fibrotic anatomy. Devices with integrated injection capability further streamline workflow by reducing instrument exchanges.

As evidence accumulates, scissor-type knives appear poised to become the dominant tools in flexible endoscopic management of ZD, although device choice should remain individualized on the basis of anatomy, operator expertise, and institutional availability.

## Adverse events of ZD endoscopic therapy and management

### Minor adverse events

Dental or lip injuries can occur, particularly with rigid devices or overtubes^1^; however, protective bite blocks and careful technique help prevent this. Transient sore throat, odynophagia, or hoarseness are expected but typically resolve within a few days.^1^ Injury to the vocal cords or recurrent laryngeal nerve has been reported in surgical series, but this is exceedingly rare in purely endoscopic cases.^12^

### Major adverse events

#### Bleeding

Given the vascularity of the pharyngoesophageal area, bleeding is a key consideration, although clinically significant hemorrhage is uncommon with modern techniques (∼1%-3%).^7^ Patients receiving anticoagulants or antiplatelet agents are usually managed by holding these medications perioperatively to mitigate bleeding risk, following the American Society for Gastrointestinal Endoscopy guidelines.^36^^,^^37^ One of the advantages of scissors-type knives (eg, ClutchCutter, SB Knife) is that they allow for pre-emptive coagulation of the grasped tissue before cutting.^38^ With needle-knife devices, many experts also prophylactically coagulate visible septal vessels before transection, either with the knife itself or with dedicated coagulation forceps.^39^

If brisk intraprocedural bleeding occurs, the first step is to maintain visualization with irrigation, followed by direct hemostasis by applying coagulation with the knife tip, deploying dedicated hemostatic forceps (eg, Coagrasper, Olympus America), or, with scissor-type devices, by grasping the vessel and applying a soft-coagulation current.^38^ Through-the-scope clips (TTSCs) are typically of limited value because placement is challenging in the confined space and may hinder completion of the procedure. Delayed postprocedural bleeding is very rare but requires urgent endoscopy for hemostasis with clipping or coagulation of the culprit vessel.^39^ With meticulous technique and modern tools, bleeding is usually minor and readily controlled endoscopically.

## Perforation and leakage

Perforations can occur if a myotomy extends too deeply into the esophageal wall during FES or if a mucosotomy created during Z-POEM fails to seal. Careful mucosal inspection at the end of the procedure is crucial.^39^ The incidence of clinically significant perforation is relatively low (∼2%-6%).^13^^,^^22^^,^^36^ The use of carbon dioxide (CO_2_) insufflation is mandatory because it is rapidly absorbed and minimizes the risk of mediastinal and subcutaneous emphysema commonly associated with the Z-POEM technique in the setting of microperforation.^24^

Some controversy exists regarding the need for incision closure after standard septotomy. Because the typical incision does not extend beyond the distal extent of the diverticulum, closure may not be required.^40^ In contrast, closure is strongly recommended after the Z-POEM technique.^41^ Standard TTSCs, applied in a “zipper” fashion, are most commonly used for incision closure.

Because delayed perforation/leakage is rare, routine hospitalization after Z-POEM is generally unnecessary, except in cases where closure was technically difficult or its reliability is uncertain. If delayed leakage occurs, it can lead to submucosal tunnel infection, mediastinitis, and mediastinal abscess formation, which can manifest as fever, sepsis, chest pain, or subcutaneous emphysema.^42^ Management is guided by the patient's clinical stability and defect characteristics. Small contained leaks may be managed conservatively with nil per os and antibiotics.^23^ Endoscopic closure is usually the first-line approach, and short-stem TTSCs are particularly well suited for Z-POEM, because their reduced tail length improves visualization and enables closely spaced clip deployment within the narrow hypopharyngeal anatomy, thereby facilitating mucosal closure. Over-the-scope clips (OTSCs), endoscopic suturing, or stent placement may be considered in select cases.^43^ Because perforations after FES or Z-POEM occur at the level of the upper esophageal sphincter, traditional esophageal stents are often poorly tolerated, given that the proximal end must be positioned in the hypopharynx. Recently, a dedicated stent with a smaller, softer proximal flange designed for placement across the upper esophageal sphincter has become available (HILZO UES; Thoracent, Huntington, NY, USA). Significant fluid collections or signs of sepsis warrant immediate endoscopic intervention, including lavage and catheter-based drainage.^7^ If advanced endoscopic therapy is unavailable or unsuccessful, surgical drainage (open or video-assisted) remains the definitive rescue option. The key to successful outcomes is early recognition. Any concerning symptoms after endoscopic myotomy should prompt immediate imaging and evaluation.

## Mediastinitis and abscess

Mediastinitis, the most feared adverse event, is exceedingly rare in the modern endoscopic era (<0.5% in large series).^11^ Prevention is paramount and involves meticulous suctioning of diverticular contents before myotomy to minimize potential contamination risk and reliable incision closure.^4^

Management of this life-threatening event requires a swift, multidisciplinary approach because clinical presentation is typically severe with high fever, chest pain, and signs of sepsis. Initial steps include broad-spectrum intravenous antibiotics and antifungals, along with urgent evaluation for drainage of any formed abscess. Although this has traditionally required open surgical drainage, advanced endoscopic rescue techniques are emerging, such as endoluminal vacuum–assisted closure (EndoVAC) therapy.^44^ This involves endoscopically placing a polyurethane sponge into the mediastinal cavity through the esophageal defect, connected to a vacuum-assisted closure device under continuous negative pressure.^44^ Active drainage of the abscess with periodic sponge changes promotes progressive cavity collapse, potentially avoiding the need for surgery.^44^ Similarly, endoscopic transmural drainage approaches can rapidly stabilize mediastinal infections.^45^

## Subcutaneous emphysema and pneumomediastinum

Insufflation-related gas dissection into soft tissues is well recognized. The routine use of CO_2_, which is rapidly absorbed, has rendered it a largely benign and self-limited event.^39^ Although minor cervical crepitus is not uncommon, clinically significant subcutaneous emphysema or pneumomediastinum is rare. The incidence in rigid endoscopy is approximately 1.3%,^10^ whereas in Z-POEM it may be higher (10%-15%); however, these cases are typically transient and clinically inconsequential.^16^

Management is guided by severity. If significant emphysema develops intraprocedurally (eg, neck swelling), the procedure is paused for 10 to 15 minutes to allow for CO_2_ absorption.^39^ If necessary, ventilator adjustments to enhance CO_2_ clearance or chest tube placement for a clinically significant pneumothorax may be used.^39^ Before the procedure, management is conservative, involving observation for respiratory compromise or infection, supplemental oxygen to expedite gas reabsorption, and pain management with resolution typically within 24 to 48 hours.^39^^,^^46^ Severe but rare adverse events, such as airway compression from a progressive pneumomediastinum or cardiac tamponade from tension pneumopericardium, have been described in the broader third-space endoscopy literature.^46^ However, the need for surgical decompression (eg, video-assisted thoracoscopic surgery or pericardial window) is exceedingly rare in the context of Z-POEM.^46^

## Aspiration and respiratory adverse events

Patients with ZD have an inherent aspiration risk from retained pouch contents that can be regurgitated or spilled into the airway, potentially leading to pneumonitis or pneumonia.^10^ Therefore, prevention is the primary goal. Beyond strict nil per os status and meticulous suctioning of the diverticulum at the start of the procedure,^4^ the most critical safeguard is definitive airway protection.^23^

Minor aspiration leading to transient pneumonitis may only require monitoring and supportive care (eg, supplemental oxygen). The development of aspiration pneumonia, presenting with fever, leukocytosis, and chest infiltrates, necessitates broad-spectrum antibiotics. Severe cases may require hospitalization, ventilatory support, and bronchoscopy to clear particulate matter. Although clinically significant aspiration is uncommon with modern protocols, it remains a critical concern, underscoring the importance of preventive airway protection.

## Prevention of adverse events

### Need for antibiotics

Preventing infection in endoscopic Zenker’s management hinges on meticulous technique, proper preparation, and vigilant postprocedure care. Routine prophylactic antibiotics are not advised; however, they may be administered if wall injury is suspected.^13^^,^^24^^,^^33^

### Anesthesia requirements

Although FES can be performed without general anesthesia, most experts favor general anesthesia with endotracheal intubation to protect the airway and minimize aspiration risk.^8^^,^^12^^,^^23^

### Endoscopic devices

A complete third-space endoscopy armamentarium should be readily available. Z-POEM is typically performed using a standard adult forward-viewing diagnostic gastroscope with a distal attachment cap (Olympus America) that typically provides sufficient septal exposure with adequate working space.^24^ Slim therapeutic gastroscopes with large working channels, such as the Fujifilm EG-840TP or Olympus GIF-1TH190, are increasingly favored in expert centers to enhance maneuverability and facilitate dissection and closure within the confined hypopharyngeal space. A dedicated ZD overtube (Cook Endoscopy, Winston-Salem, NC, USA) exists but is rarely used because of positioning challenges.

Incision closure can be achieved in the majority of cases using TTSCs.^43^ In situations where TTSC-based closure is technically challenging, alternative approaches such as OTSCs or the X-Tack system (Boston Scientific) can be used.^43^^,^^47^ In contrast, the OverStitch endoscopic suturing system (Boston Scientific) is generally not well suited for defect closure in the tight hypopharyngeal space, because of limited maneuverability and working space.

### Learning curve and case selection

Virtually all patients with symptomatic ZD are candidates for flexible endoscopic treatment. However, outcomes are linked to a significant learning curve.^4^ Novice operators should begin with treatment-naïve cases, whereas salvage cases with fibrosis should be reserved for advanced operators.^4^ FES with scissor-type knives is generally more intuitive for therapeutic endoscopists, whereas Z-POEM requires mastery of third-space endoscopy and should be performed only by those trained in third-space techniques, including endoscopic closure.^3^^,^^23^ Mentored exposure and animal model training can help shorten the learning curve, and scissor-type knives provide greater control than freehand needle-knife techniques.

Although Z-POEM typically takes longer than septotomy (30-60 vs 15-30 minutes), procedure times decrease with experience.^2^^,^^12^^,^^48^ High success rates combined with low adverse event rates have enabled many centers to perform these procedures on an outpatient basis.^49^

### Postprocedure care

Traditionally, patients undergoing Z-POEM were admitted overnight, kept nil per os, and underwent next-day contrast esophagography, with lengths of stay comparable to FES.^2^^,^^19^^,^^23^ Current practice favors same-day discharge in most patients without significant adverse events who demonstrate an early negative esophagogram, stable vital signs, adequate pain control, tolerance of liquids, and reliable social support.^50^

## Future directions

### Current evidence gaps and the need for standardization

Despite compelling data from large retrospective series and meta-analyses, the current evidence base lacks high-quality, prospective, multicenter trials directly comparing modern FES, Z-POEM, and rigid septotomy.^2^ Such trials are essential to assess long-term durability, patient-reported outcomes, and cost-effectiveness.^12^ Technical heterogeneity also persists, with numerous variations in tunneling approaches, device selection, and closure techniques. To ensure consistent high-quality outcomes as adoption expands, future efforts should prioritize standardized procedural guidelines and structured training pathways with defined competency metrics.

### The horizon: refining techniques and defining value

Future innovation will likely focus on 3 key areas. First, device development will continue, with emerging bipolar energy platforms and the potential for a dedicated flexible endoscopic stapler to further optimize hemostasis and procedural efficiency. Second, adverse event management protocols require standardization; although techniques such as EndoVAC are promising for leak management, prospective studies are needed to formalize their application. Moreover, the optimal antibiotic regimen and duration after a Zenker’s perforation remain unclear, with most protocols recommending broad coverage for at least 7 days; however, no consensus exists.^39^

Finally, the field must move beyond measuring only technical success. Future research should prioritize patient-centered and economic outcomes, rigorously comparing quality of life, nutritional improvement, and cost-effectiveness across approaches. Generating this high-level data will be crucial to solidifying the role and value of these innovative techniques in the modern treatment algorithm for ZD.

## Conclusions

Endoscopic therapy has transformed the management of ZD, replacing open surgery with minimally invasive approaches that achieve comparable clinical success and markedly less morbidity. Z-POEM enables complete myotomy under direct visualization, with enhanced precision and safety compared with direct flexible septotomy. Vigilance, structured training, and standardized protocols are essential to prevent and manage rare but serious adverse events. The field now stands at an inflection point, where further technical refinement, rigorous comparative trials, and patient-centered outcomes will determine the lasting value of these advances.

## Patient consent

No individual patient data are included in this manuscript; therefore, informed consent for publication was not applicable.

## Ethics statement

This article is a narrative review and does not involve any original research with human participants or animals. As such, Institutional Review Board approval was not required.

## Disclosure

All authors disclosed no financial relationships.
